# Perceived barriers and facilitators to the feasibility and acceptability of a peer-to-peer (P2P) HIV self-testing distribution model among adolescents and young people aged 15–24 years in Uganda: a qualitative study

**DOI:** 10.1136/bmjph-2026-005074

**Published:** 2026-08-05

**Authors:** Denis Ndekezi, Richard Muhumuza, Jackline Namara, Rehema Nagawa, Esther Awino, Felix Rutaro, Joanita Nassali, Andrew Sentoogo Ssemata

**Affiliations:** 1 Department of Population Health, London School of Hygiene & Tropical Medicine, London, UK; 2 Social Determinants of Health, MRC/UVRI and LSHTM Uganda Research Unit, Entebbe, Uganda; 3 Data and Statistics, MRC/UVRI and LSHTM Uganda Research Unit, Entebbe, Uganda

**Keywords:** HIV, Adolescent, Qualitative Research

## Abstract

**Introduction:**

Peer-to-peer HIV self-testing (HIVST) has shown promise in addressing barriers to HIV testing challenges by leveraging social networks and peer influence to promote health-seeking behaviours.

**Objective:**

To explore the perceived feasibility and acceptability of the peer-to-peer HIVST distribution model among adolescents and young people aged 15–24 years in Uganda.

**Methods:**

We conducted a descriptive exploratory qualitative study in Wakiso District, Uganda. Data were collected through six focus group discussions (n=68) and 14 in-depth interviews, including interviews with peer leaders. Participants were purposively recruited through community meetings with support from Village Health Teams. Discussions explored perceptions of peer-to-peer HIVST distribution, including potential barriers and facilitators. Interviews were audio-recorded, transcribed verbatim, translated into English and analysed using thematic analysis.

**Results:**

Participants perceived the peer-to-peer HIVST distribution model as both feasible and acceptable. Key facilitators of feasibility included peer trust, structured training, community-based delivery and integration with existing health structures. Perceived barriers included logistical constraints, inadequate supervision, kit storage challenges and concerns about equitable reach. Acceptability was driven by trust, confidentiality, emotional support, autonomy and the relatability of peer distributors. However, concerns about gossip, stigma, confidentiality breaches and the emotional burden placed on peer distributors were identified as potential barriers to sustained acceptability.

**Conclusion:**

Peer-to-peer HIVST distribution was perceived as a feasible and acceptable approach for increasing HIV testing among adolescents and young people. Future implementation efforts should strengthen peer training and supervision, address confidentiality and stigma concerns, provide psychosocial support for peer distributors, and ensure equitable access for socially marginalised adolescents.

**WHAT IS ALREADY KNOWN ON THIS TOPIC**

- HIV self-testing (HIVST) is acceptable among adolescents and young people in sub-Saharan Africa and can overcome barriers such as stigma, lack of privacy and negative experiences with facility-based testing; however, evidence on peer-to-peer distribution models among adolescents in Uganda remains limited, particularly regarding implementation challenges and social dynamics.

**WHAT THIS STUDY ADDS**

- This study shows that peer-to-peer HIVST distribution is perceived by adolescents in Ugandan fishing communities as both practical and socially appropriate, driven by peer trust, emotional safety and autonomy. It also reveals previously underexplored challenges, including confidentiality risks, emotional burden on peer distributors, logistical constraints and potential exclusion of socially isolated adolescents.

**HOW THIS STUDY MIGHT AFFECT RESEARCH, PRACTICE OR POLICY**

- The findings highlight the need for peer-led HIVST models to incorporate structured training, ongoing mentorship, emotional support mechanisms and deliberate equity strategies. Integrating peer distributors within existing health systems and community structures may strengthen sustainability, safeguard confidentiality and support scale-up of youth-friendly HIV testing services in high-prevalence settings.

## Introduction

 The United Nations defines adolescents as individuals aged 10–19 years and young people as those aged 10–24 years.^1^ In this study, adolescents and young people (AYP) refers to individuals aged 15–24 years. Adolescents and young people continue to face disproportionate risks in the HIV epidemic. Globally, there were approximately 1.3 million new HIV infections in 2023, with sub-Saharan Africa remaining the epicentre. The region accounts for over 4500 new HIV infections every week among adolescent girls and young women aged 15–24 years.^2^ Uganda continues to face a significant HIV burden among AYP. According to recent national estimates, approximately 1.4 million people were living with HIV in Uganda in 2023, with AYP remaining disproportionately affected by new infections and gaps in HIV testing services.^3^

Earlier estimates indicated that approximately 53 000 new HIV infections occurred in 2019, accounting for nearly two-thirds of all new infections nationally, with districts such as Wakiso, Kampala and surrounding areas contributing substantially to the epidemic.^4^ Findings from the Uganda Population-based HIV Impact Assessment showed that only 37.9% of males and 48.7% of females aged 15–19 years had ever tested for HIV and received their results, indicating substantial unmet testing needs among adolescents.^5^ These gaps are particularly concerning in fishing communities and other high HIV-burden settings, where stigma, population mobility, limited access to youth-friendly services, fear of judgement and concerns about confidentiality continue to hinder uptake of HIV testing services.^6–8^ These challenges highlight the need for innovative, youth-friendly approaches that address the multiple factors influencing HIV testing among AYP, including individual, interpersonal, community and health-system barriers. Understanding how these factors may influence the feasibility and acceptability of alternative testing approaches is critical for improving HIV testing uptake and linkage to care among AYP.

HIV self-testing (HIVST) has emerged as a promising strategy to increase testing uptake, offering greater privacy, autonomy and convenience.^9,10^ However, recent evidence from Uganda suggests that AYP continue to express concerns regarding confidentiality, stigma, disclosure of results and emotional preparedness when engaging with HIVST.^11^ While HIVST is generally acceptable among adolescents, little is known about the feasibility and acceptability of peer-to-peer (P2P) HIVST distribution models in Uganda. Understanding how adolescents perceive this delivery approach is important for informing the design and implementation of youth-friendly HIV testing strategies.

This study directly addresses this gap by exploring adolescents’ and young people’s perceptions of the potential feasibility and acceptability of a proposed P2P HIVST distribution model, including factors that may facilitate or hinder its implementation.

The P2P HIVST model leverages trained AYP to distribute HIVST kits to their peers through trusted social networks.^12,13^ This peer-driven approach builds on existing social influence and confidentiality to promote HIV testing and early linkage to care.^14^ Similar peer-led interventions, such as those under the DREAMS initiative, have successfully combined HIV education, community-based testing and empowerment, resulting in a 30% reduction in HIV incidence among adolescent girls and young women in high-prevalence areas.^15^ However, potential challenges such as social harms, stigma, weak linkage to care, inadequate peer training and logistical constraints may hinder effective implementation.^16–18^ Ensuring confidentiality, community trust and sustainability is essential for scaling up this model in resource-limited settings.^19^

In this study, perceived feasibility refers to adolescents’ and young people’s views on whether the P2P HIVST model could be practically implemented within Uganda’s resource-constrained and socially dynamic contexts, including considerations of training, resources, supervision, accessibility and integration with existing systems. Perceived acceptability relates to participants’ views on the appropriateness of the model, including trust, comfort, confidentiality, willingness to engage and perceived social acceptability.^20^ Given the limited evidence on P2P HIVST distribution among adolescents in Uganda, this study adopted an exploratory qualitative approach to understand adolescents’ perceptions of the model’s feasibility and acceptability and to identify factors that may influence its implementation. By examining these dimensions, this study contributes evidence to inform the design, adaptation and scale-up of peer-led HIV testing strategies that can strengthen health equity and accelerate HIV prevention among Ugandan adolescents.

## Materials and methods

This study employed a descriptive exploratory qualitative design to explore adolescents’ and young people’s feasibility and acceptability of a proposed P2P HIVST distribution model and to identify perceived barriers and facilitators that could influence its implementation in Uganda. This study was conducted between March 2021 and February 2022 and was nested within the HIV Self-Testing among Adolescents in Zambia and Uganda study, a mixed-methods study that explored the acceptability and social implications of P2P HIVST among AYP aged 15–24 years in Uganda and Zambia.^13^

The study was conducted in three fishing communities—Kigungu, Gerenge and Nakiwogo—in Wakiso District, Central Uganda. Wakiso is Uganda’s most populous district, with about 22% of its population aged 15–24 years,^21^ and has a high HIV burden.^5^ The selected communities have large populations of AYP engaged in fishing and hospitality-related work, occupations associated with high mobility and increased HIV vulnerability.^22^ At the time of the study (2021–2022), HIVST was not widely available in Uganda, making these communities an appropriate setting to explore perceptions of the feasibility and acceptability of P2P HIV self-testing distribution.^22^

### Study population

This study focused on AYP, aged 15–24 years, residing in or around fishing communities in Wakiso District, Uganda. Participants included both in-school and out-of-school AYP, as well as peer leaders, to ensure diverse perspectives on P2P HIVST. Both males and females were eligible to participate. The term ‘peer’ referred to community members of a similar age, social status or life circumstances. Participants were eligible regardless of HIV status, as no HIV testing or screening was conducted as part of the study.

### Selection and recruitment

Participants were purposively recruited through the village information meetings and peer mobilisation activities conducted with the support of the community health extension workers (CHEWs), formerly Village Health Teams (VHTs). The CHEWs mobilised eligible AYP from both in-school and out-of-school settings to attend community meetings, where the study team provided information about the study and invited interested individuals to participate. Recruitment aimed to achieve variation in age, sex, schooling status and community roles, including peer leaders, to capture a broad range of perspectives and experiences.

### Data collection procedure

Before data collection, participants received a standardised explanation of HIVST, including its purpose, use, result interpretation and the proposed P2P distribution model, to ensure a common understanding before discussing its feasibility and acceptability. Data were collected through six focus group discussions (FGDs; n=68 participants) and 14 in-depth interviews (IDIs), including four interviews with peer leaders. All interviews and discussions were conducted face-to-face by four trained social scientists (two male and two female) using a piloted semi-structured interview guide (online supplemental files 1 and 2). To protect confidentiality in group settings, participants were reminded to respect privacy and not share group discussions outside the session. Sessions were held in private locations, and facilitators emphasised confidentiality at the outset. The guide explored perceptions of the feasibility and acceptability of P2P HIVST, including potential barriers and facilitators, preferred distribution approaches, access points and preferences for oral versus blood-based HIV self-test kits.

Participants for the IDIs were recruited separately from those participating in FGDs. FGDs were used to capture shared community perspectives and social norms, while IDIs provided more detailed individual accounts on this sensitive topic. Data from both methods were analysed together to provide a comprehensive understanding of participants’ views. Interviews and discussions lasted between 50 and 90 min and were conducted in participants’ preferred language (English or Luganda) in private locations within the study communities. Written informed consent was obtained from all participants, and assent with parental or guardian consent was obtained for participants under 18 years of age. The sample size was guided by the principle of data saturation, whereby recruitment continued until no new themes or insights emerged. This approach is consistent with qualitative research guidance suggesting that thematic saturation can often be achieved within a relatively small number of interviews and FGDs, depending on the study aims and participant diversity.^23,24^

### Patient and public involvement

The study involved adolescents, young people and peer leaders throughout the research process. Peer leaders reviewed the interview guides and suggested additional topics for interviews and FGDs. At the end of the study, three community dissemination meetings were held, one in each study community, with over 50 participants attending each meeting. Participants reviewed the findings and confirmed that they reflected their experiences and perspectives. No major discrepancies or new themes were identified.

### Data management and analysis

Audio recordings were transcribed verbatim, translated into English, and securely stored to maintain confidentiality. Data were analysed using a hybrid thematic analysis approach informed by Braun and Clarke.^25^ Following familiarisation with the transcripts, a preliminary coding framework was developed. Deductive codes were derived from the study objectives and interview topics, while inductive coding identified concepts emerging from the data. Three researchers participated in coding, with two primary coders independently coding initial transcripts and a senior researcher facilitating consensus discussions. The coding framework was iteratively refined and systematically applied to all transcripts. Related codes were grouped into categories, which were then compared across transcripts and refined into broader themes and sub-themes through iterative discussions among the research team. Patterns were examined across participant characteristics, including age and sex, to explore potential differences in perceptions. However, the analysis focused on identifying shared themes across participants rather than comparing demographic subgroups. Throughout the analysis, reflective memos and regular team discussions were used to support reflexivity and consistency. The findings were interpreted in relation to participants’ perceptions of feasibility and acceptability. Themes and sub-themes were iteratively reviewed and refined to ensure they accurately reflected participants’ experiences and were aligned with the study objectives. Representative quotations were selected to illustrate the findings. The Standards for Reporting Qualitative Research (SRQR) guidelines informed the reporting of this study (online supplemental file 3).

## Results

A total of 82 participants took part in both the IDIs and FGDs, primarily adolescents and young adults aged 15–24, with the largest proportion falling in the 18–24 age group (79.3%). The sample included slightly more females (53.7%) than males and was predominantly composed of students (59.8%) (table 1).

**Table 1:** Participant demographic characteristics

Category	Sub-category	Frequency	Percentage
Age group	15–17	17	20.7
	18–24	65	79.3
Sex	Male	38	46.3
	Female	44	53.7
Education level	Primary	14	17.1
	Secondary	60	73.2
	University	8	9.8
Occupation	Student	49	59.8
	Fishing-related work	18	22.0
	Other occupations*	3	3.7
Marital status	Single	80	97.6
	Married	2	2.4
Religion	Muslim	30	36.6
	Christian**†**	52	63.4
Ethnicity	Muganda	30	36.6
	Other	52	63.4

* Includes self-employed, casual workers, hairdressers, potters, builders, artists and other occupations.

† Includes Catholic, Protestant, and Born-Again Christians.

The P2P HIVST distribution model was perceived as both feasible and acceptable among AYP. Feasibility was supported by adequate training, peer trust, familiar community settings and integration with existing health structures, while logistical and resource constraints, storage concerns, equity issues and weak supervision were identified as potential barriers. Acceptability was enhanced by trust, confidentiality, emotional support, autonomy and alignment with adolescent preferences. However, concerns about gossip, stigma, the emotional burden placed on peer distributors and uncertainty regarding long-term sustainability could limit acceptability (online supplemental file 4).

### Perceived feasibility of the P2P HIVST distribution model

#### Peer distributor capacity and support

Adolescents perceived the feasibility of the P2P HIVST distribution model to be linked to the capacity and support of peer distributors. They highlighted training, mentorship, and social trust as critical enablers. Structured, community-based training was seen as essential for equipping peers with the knowledge and confidence to distribute kits accurately and provide guidance to others. One participant explained:

Get a responsible adolescent and train him or her to understand how to use it. He tensioncan train his peers and distribute the kits. (Female IDI, 17 years)

Participants also emphasised peer trust and solidarity as a facilitator. Familiarity and shared experiences among peers were perceived to reduce anxiety around HIV testing and increase willingness to participate:

Adolescents are confident among themselves … if I get it from a friend, we would even test together. (Male IDI, 17 years)

However, several perceived barriers could limit feasibility. Peer distributors reported emotional and social pressures, including concerns about managing friends’ reactions and maintaining relationships after a positive result:

When I give a peer a kit … what if they blame me? Will they be okay? … It’s emotionally draining. (Female IDI—Peer leader)

Additionally, adolescents perceived a strong need for ongoing supervision and mentorship. Without regular check-ins and refresher support, peer distributors feared mistakes, loss of motivation or inconsistent distribution:

Even as peer leaders we need refresher training and follow-up; otherwise, many will lose interest or make mistakes. (Male IDI—Peer leader)

Adolescents perceived that the feasibility of the P2P HIVST model depends on well-prepared, supported, and trusted peer distributors, with structured training and continuous mentorship as essential mechanisms to sustain effective implementation. These perceptions suggest that implementation strategies should prioritise building peer capacity and providing ongoing support to address both practical and emotional challenges.

#### Logistical and operational considerations

Adolescents perceived several logistical and operational factors as critical to the feasibility of the P2P HIVST distribution model. Familiar, community-based settings and collaboration with local health structures such as VHTs and youth corners were seen as important enablers. These arrangements were perceived to make distribution more practical, enhance trust and strengthen linkage to care:

If peer leaders work with healthcare workers or VHTs, it becomes easier because they already know where to refer us. (Male IDI, 19 years)

Participants also highlighted the value of discreet and respectful kit delivery, which was perceived to encourage uptake and protect confidentiality. Adolescents emphasised that visible or public distribution could lead to stigma or embarrassment, whereas private delivery by peers increased comfort and willingness to test.

Despite these facilitators, several perceived barriers threatened feasibility. Transport and resource limitations, especially in remote or island communities, were identified as major challenges. Adolescents reported that long distances, lack of transport and the costs associated with reaching peers could restrict equitable access:

There are some places which you cannot reach on foot; it requires a motorbike which you can’t afford. (Male IDI—Peer leader)

Storage and handling of HIVST kits was another perceived barrier. Participants noted that overcrowded or unhygienic home conditions could compromise kit integrity, and improper handling by peer distributors could affect accuracy and trust in the results:

You can’t just keep the kit anywhere … if the place is dirty or hot, it might get spoiled. At home we don’t have much space, so it needs to be kept somewhere safe and private, or people might mess with it. (Female IDI, 17 years)

Weak monitoring and coordination were viewed as potential threats to the consistency and accountability of the distribution process. Adolescents perceived that without structured supervision and follow-up, peer distributors might make errors, deliver kits unevenly or lose motivation:

It is possible for peer leaders to distribute the kits, but the only challenge is that some peers are lazy if not supervised. The best thing would be stationing in one place or having a system to monitor the peer distributors. (Male IDI, 20 years)

Adolescents perceived that the feasibility of the P2P HIVST model depends on practical adaptations and support mechanisms, including accessible distribution settings, integration with local health structures, transport solutions, proper kit storage and structured monitoring.

#### Reach, equity and system integration

Adolescents perceived that peer-led HIVST distribution enhanced accessibility, particularly for those who were uncomfortable engaging with formal health services. Receiving kits from trusted peers allowed adolescents to test privately and avoid potentially judgemental interactions with healthcare workers, making the process feel safer and more approachable:

Peer distribution will work well for adolescents because many times we are less interested in going to the village health teams … It feels good for the peer to give me the kit to test in private. (Female IDI, 15 years)

Integration with schools and existing community networks was perceived to further support reach and scalability, especially for in-school adolescents who could easily access kits through peer networks. Familiar social environments and daily peer interactions were seen as important enablers for uptake.

However, several perceived barriers could limit equity and long-term effectiveness. Adolescents noted that out-of-school, isolated or socially disconnected peers risked being excluded from the distribution network, particularly if peer distributors prioritised friends or close contacts:

Some peers will end up giving only their friends … one can keep some for girlfriends. (Female IDI—Peer leader)

Participants also expressed doubts about sustainability, questioning whether the programme would be consistently implemented or supported over time. Past experiences with incomplete interventions contributed to cautious perceptions about follow-through and programme continuity:

I doubt that this peer distribution programme will ever be implemented here … we don’t see any action (Male IDI—Peer leader)

### Perceived acceptability of the P2P HIVST distribution model

#### Social and emotional dynamics

Adolescents widely perceived the P2P HIVST distribution model as highly acceptable, primarily due to the sense of emotional safety, trust and shared experience it provided. Receiving kits from peers was considered less intimidating than facility-based services and allowed adolescents to test privately, maintaining confidentiality and reducing anxiety. Many participants described peers not only as distributors but as trusted confidants capable of offering moral and practical support before, during and after testing:

Your fellow peer can keep it as a secret … if I tested positive, I can lean on them for support. (Female FGD, 15–18 years)

When you look at your agemate giving you the test kit, you get the confidence to test. It feels better than someone older than you who may not understand how you feel*.* (Male IDI, 17 years)

The model was also perceived to enhance HIV testing uptake, especially among adolescents who were hesitant to engage with traditional services. Participants noted that peer-led delivery allowed for practical guidance on kit use, interpretation of results and safer behaviours, which could empower peers and reduce harmful reactions:

Yes, the peer distribution model will increase the numbers of adolescents testing because some adolescents will see it as a new approach and as something new and nice they can easily adopt. (Male IDI, 17 years)

I would personally feel so happy to receive the kit from a fellow peer and would want them to practically demonstrate how it is used so I can interpret the results correctly*.* (Female FGD, 15–18 years)

A key facilitator of acceptability was autonomy and alignment with adolescent values. Adolescents appreciated the non-coercive, choice-driven nature of the model, where peers served as facilitators rather than authority figures:

I’m okay with it because it is not by force and everyone wants to know his or her status*.* (Female FGD, 15–18 years)

The model’s social fit further reinforced acceptability. Familiarity with the peer distributor, social closeness and informal delivery were important in building trust and comfort, which adolescents felt was often lacking in interactions with healthcare workers:

The peer model will be a good strategy because I feel more confident picking it from my friend than the healthcare workers who are rude on us when we go for testing at the facilities. (Female IDI, 17 years)

When we talk to them about the kits, this can help them. They trust us so much. Actually, among adolescents that method would be very effective. (Male IDI, Peer Leader)

Receiving kits from peers was also described as empowering, providing opportunities for discussion and clarification, which reduced anxiety and promoted informed decision-making:

When you take the kit to a friend you explain to them about all the procedures and how to interpret the results. This will empower many of our friends to test for HIV … there are some questions you can feel free to ask them compared to an older person. (Female GD, 15–17 years)

#### Barriers to acceptability

Despite these facilitators, several barriers to acceptability were noted. Confidentiality and gossip concerns were prominent, with adolescents fearing that peers might make assumptions about their HIV status or pressure disclosure:

Most of them like gossiping so they may talk about my results. It is hard to trust friends. (Female GD, 18–24 years)

When he brings it to you, he would want to know the results … I would want someone who doesn’t know me to distribute this kit to me. (Female GD, 18–24 years)

Peer distributors also experienced role strain, navigating complex emotional, social and logistical pressures. Delivering kits could be physically demanding in hard-to-reach areas and emotionally taxing, particularly when friends tested positive or community stigma was anticipated:

There are some places which you cannot reach on foot; it requires a boda boda which you can’t afford … You can worry on how to handle the kits in that situation when you have no transport means. (Male IDI—Peer leader)

When I give a peer a kit … I might be worried about their reaction if they test positive. It’s emotionally draining, seeing your friend struggle with their results. (Female IDI—Peer leader)

Since people in my area get to know that my friend tested positive with the kit I distributed, they might think I’m ‘the HIV person’ now. It’s a lot of pressure to us now. (Male IDI—Peer leader)

## Discussion

This study explored adolescents’ and young people’s perceptions of the feasibility and acceptability of a P2P HIVST distribution model in Uganda. The findings suggest that the model is perceived as both acceptable and feasible, with trust, familiarity and shared experiences acting as key enablers. However, concerns regarding confidentiality, stigma, logistical constraints, equitable access and sustainability highlight important considerations for implementation and scale-up.

The high perceived acceptability of the model reflects adolescents’ desire for emotionally safe, non-judgmental spaces when confronting sensitive health issues—needs often unmet in formal healthcare settings. Peer delivery appears to tap into shared social identity and relational trust, enabling adolescents to feel more comfortable accepting HIVST kits. This aligns with broader literature showing that HIVST among young people in sub-Saharan Africa is facilitated by privacy, autonomy and the opportunity to avoid traditional facility-based barriers.^26^ To maintain this acceptability, peer distributors should receive comprehensive training in confidentiality, ethical conduct and emotional coping, ensuring that adolescents continue to feel safe and respected during distribution. Continuous mentorship and support through youth-friendly services reinforces these skills, preventing burnout and ensuring high-quality delivery.^26^

Feasibility, however, depended heavily on system and peer-distributor capacity training, mentorship, logistical integration and proper handling. Adolescents stressed that peer leaders must be trained, supported and part of the health structure to distribute kits credibly. This is consistent with implementation science findings that peer-led interventions require both relational trust and institutional scaffolding to function well.^27^ To strengthen feasibility, peer distributors should be formally linked with health structures such as VHTs and youth-friendly clinics. This ensures oversight, builds credibility, facilitates referral pathways and supports long-term sustainability of HIVST delivery.

A particularly novel insight is the psychosocial burden experienced by peer distributors emotional strain in assisting friends who may test positive, fear of blame and anxiety about confidentiality. This underscores that feasibility is not simply about material and logistical readiness, but also the emotional and ethical preparedness of peers a dimension less represented in earlier HIVST literature. In this way, our findings extend the discourse beyond uptake and acceptability to sustained delivery capacity and well-being of implementers. Implementing emotional support mechanisms, including rotation of peer distributors and regular debriefing sessions, can reduce stress, prevent burnout and sustain the effectiveness and long-term viability of peer-led HIVST delivery. The tension between trust and confidentiality emerged as a central theme. While adolescents preferred receiving HIVST kits from trusted peers, familiarity simultaneously created concerns about gossip, unwanted disclosure and stigma. This paradox has been noted in previous reviews of HIVST and peer distribution.^28^ Similar concerns have recently been reported among AYP in Uganda, where fears of disclosure, social judgement and emotional consequences influenced perceptions of HIV self-testing.^11^ Our data show how this tension plays out among adolescents, suggesting peer models must carefully manage relational closeness and confidentiality safeguards.

The equity dimension also requires attention: the model’s reliance on peer networks may inadvertently exclude adolescents who are out-of-school, socially isolated or minimally connected. This mirrors broader concerns that community-led and peer-led programmes can replicate existing social inequalities rather than overcome them.^29^ To ensure equity, peer delivery must be complemented by deliberate mapping of underserved youth and inclusive distribution strategies.

### Strengths and limitations

A key strength of this study was its youth-centred approach, which directly engaged AYP in exploring the feasibility and acceptability of a P2P HIVST model. This ensured that the findings reflected the lived experiences, priorities and social realities of the target population. The inclusion of both in-school and out-of-school adolescents captured diverse perspectives that are often under-represented in health research. In addition, the use of both FGDs and IDIs strengthened the credibility of the findings through methodological triangulation.

This study also has several limitations. As a qualitative exploratory study conducted in selected fishing communities, the findings provide an in-depth understanding of participants’ perspectives within this context. While the findings may be transferable to similar settings, their applicability to other populations should be considered in light of contextual differences. Although trained interviewers, standardised procedures, and participant validation through community dissemination meetings were used to enhance credibility, social desirability bias may have influenced some responses, particularly during FGDs. Recruitment through CHEWs and VHTs may have underrepresented adolescents who were socially isolated or less connected to community networks, potentially limiting the range of perspectives captured. The study also did not include the views of caregivers or health facility staff, which may have provided additional insights into implementation and health-system integration. Furthermore, feasibility was explored through participants’ perceptions rather than actual implementation; therefore, kit uptake, fidelity and linkage to care could not be assessed. The study was also not guided by a specific implementation framework, as the aim was to allow themes to emerge from participants’ accounts. Future implementation studies may benefit from applying established frameworks to further examine acceptability, feasibility and implementation processes. Finally, although reflexive discussions were held throughout the analysis process, researcher positionality was not examined in depth and may have influenced data collection and interpretation.

## Conclusion

The P2P HIVST distribution model was perceived as both feasible and acceptable among AYP in Uganda. Key enablers included peer trust, shared experiences, structured training and mentorship, and integration with existing community and health-system structures. These factors were viewed as essential for promoting confidence, accessibility and engagement with HIVST. However, several barriers may limit successful implementation, including concerns about confidentiality, stigma, emotional burden among peer distributors, logistical and resource constraints, and the potential exclusion of socially marginalised adolescents. Addressing these barriers through comprehensive peer training, ongoing supervision, clear referral pathways, equitable distribution strategies and psychosocial support mechanisms will be critical for effective implementation.

The findings suggest that P2P HIVST distribution has the potential to expand youth-friendly HIV testing services in Uganda. Future pilot implementation studies should build on the identified enablers while addressing the barriers to ensure that the model is effective, equitable and sustainable in resource-limited settings.

## Supplementary material

10.1136/bmjph-2026-005074 online supplemental file 1

10.1136/bmjph-2026-005074 online supplemental file 2

10.1136/bmjph-2026-005074 online supplemental file 3

10.1136/bmjph-2026-005074 online supplemental file 4

## Data Availability

All data relevant to the study are included in the article or uploaded as supplementary information.
