# Supplementary material for: Perceived barriers and facilitators to the feasibility and acceptability of a peer-to-peer (P2P) HIV self-testing distribution model among adolescents and young people aged 15–24 years in Uganda: a qualitative study
Source: BMJ Public Health. 2026 Aug 5;4(3):e005074. doi: 10.1136/bmjph-2026-005074 (PMC13448566; doi:10.1136/bmjph-2026-005074)
Supplement: Supplementary file 1 [file bmjph-4-3-s001.docx]

**Supplemental Material 1: In-depth interview guide for Adolescents and Yong People**

**Qualitative Interview Guide**

**Background information**

- Please tell me a little bit about yourself? (hobbies, friends, where you hangout for fun)
- Tell me about the last time you tested for HIV. Why did you decide to test for HIV? Where did you test for HIV? What was your experience of testing for HIV? If NEVER tested for HIV. What are the reasons you have never tested for HIV?

**HIV RISK PERCEPTIONS**

- How do you think young people that you know are at risk of contracting HIV?
  Probe: which group of people do you think are more at risk: sex, gender, age?
- How do you think you are/ are not at risk of contracting HIV?
- Please tell me about ways that young people (including yourself) are using to protect against HIV.
- What are the current HIV testing services available in this community?

***Probe*** *what is the perception and level of acceptance and use of the existing services by adolescents and young people (AYP)?*

1. Please share with me what you know or have heard about HIVST and HIVST procedures. ***Probe*** *where they heard it from, their thoughts about HIVST*
2. What are your views about HIVST? ***Probe*** *for comparison between current routine HIV testing and HIVST?* where to get the kits, price of the kits, ability to read the correct results, who to distribute them, feasibility of young people adapting self-testing
3. Please share with some of the perceptions that other young people like you have about self-testing
4. When made available, where and how do you think HIVST kits should be accessed and distributed? ***Probe*** *for location i.e. facilities, vendors, peers, clinic, pharmacy, mobile clinics and community centres.*

***Probe*** *for reasons for the selected point of dissemination. Probe for person to disseminate.*

*Where would you recommend a friend to get HIVST kits?* ***Probe*** *Why?*

5. *What are your feelings about receiving them from peers or using a peer to peer distribution model?* ***Probe*** *best ways to share information among peers and adolescent and the facilitators and barriers of using peer distributors*
6. What are some of the motivations/facilitators of self-testing?
7. Would you be willing to pay for the Kit? How much would you be willing to pay for it and why? ***Probe*** *what would motivate you to pay?*
8. What would be the possible challenges associated with using and distributing HIV self-test kits? Or Please share with me some of the barriers to self-testing among young people. **Probe** for counselling needs, fear of the needle prick.

**Probe:** Suggest -what can be done to overcome these barriers?

9. What aspects would encourage you to take up HIVST? ***Probe*** Potential for a dramatic increase in knowledge of HIV status, increased confidentiality, increased convenience, Autonomy and empowerment).
10. What are your concerns about HIVST?

***Probe*** *Greater potential for inaccurate results, Psychological danger when decoupling testing and counselling, Greater difficulty ensuring referral to treatment and care, Potential unethical use of HIV self-testing, Self-testing as justification for unprotected sex, Concern for safe disposal of biohazard materials)*

11. [Demonstrate for participant the use of the OraQuick kit] then ask - What is your comment on the design of the kit and the testing procedures? ***Probe*** *if this is something that would be acceptable to AYP.*
12. What are your perception /opinions on test accuracy? ***Probe*** *How would you perceive the results, what would you do if the result was negative positive, unclear or you are uncertain?*
13. Is there anything else you would love to share with me about what we have been discussing or HIV testing in general?

**Thank you for accepting to participate in this research study**
