# Supplementary material for: Perceived barriers and facilitators to the feasibility and acceptability of a peer-to-peer (P2P) HIV self-testing distribution model among adolescents and young people aged 15–24 years in Uganda: a qualitative study
Source: BMJ Public Health. 2026 Aug 5;4(3):e005074. doi: 10.1136/bmjph-2026-005074 (PMC13448566; doi:10.1136/bmjph-2026-005074)
Supplement: Supplementary file 3 [file bmjph-4-3-s004.docx]

**Supplemental Material 4: Summary of Key emerging themes influencing the feasibility and acceptability of the Peer-to-Peer HIVST distribution model**

|  | **Enabling Factors** | **Barriers** |
| --- | --- | --- |
| **Feasibility** | **Adequate training and support** – Training and mentorship were viewed as essential for preparing peers to distribute and explain HIVST kits confidently. *“Get a responsible adolescent and train him or her to understand how to use it. He can train his peers and distribute the kits.”* (Female IDI, 17 years) | **Logistical and resource limitations** – Limited transport and lack of resources affected reach in remote areas. *“There are some places which you cannot reach on foot; it requires a motorbike which you can’t afford.”* (Male IDI – Peer leader) |
|  | **Peer trust and solidarity** – Strong social bonds and shared experiences increased feasibility and comfort. *“Adolescents are confident among themselves. If another person brought the kit to me, I would even be reluctant, but if I get it from a friend, we would even test together.”* (Male IDI, 17 years) | **Storage and handling issues** – Poor home conditions could damage kits or compromise integrity. *“You can’t just keep the kit anywhere… if the place is dirty or hot, it might get spoiled.”* (Female IDI, 17 years) |
|  | **Familiar community training settings** – Local and informal training environments enhanced participation. *“We can have community training for adolescents with peer educators… in the village meetings so that we know who will distribute the kits.”* (Female IDI, 15 years) | **Equity concerns** – Risks of favoritism or exclusion of isolated adolescents. *“Some peers will end up giving only their friends and leave out others… one can keep some for girlfriends.”* (Female IDI – Peer leader) |
|  | **Integration with existing community and health structures** – Collaboration with VHTs and youth-friendly clinics strengthened scalability and linkage to care. *“If peer leaders work with healthcare workers or VHTs, it becomes easier because they already know where to refer us when we need help.”* (Male IDI, 19 years) | **Weak coordination and supervision** – Lack of consistent monitoring could lead to errors and reduced motivation. *“Even as peer leaders we will need refresher training and follow-up; otherwise, many will lose interest or make mistakes.”* (Male IDI – Peer leader) |
| **Acceptability** | **Peer trust, comfort, and confidentiality** – Peers were seen as relatable and trustworthy, enhancing emotional safety. *“Your fellow peer can keep it as a secret… if I tested positive, I can lean on them for support.”* (Female FGD, 15–18 years) | **Confidentiality and gossip concerns** – Fear of peers disclosing |
|  | **Autonomy and empowermen**t – The non-coercive, youth-driven nature of the model increased acceptability. “I’m okay with it because it is not by force and everyone wants to know his or her status.” (Female FGD, 15–18 years) | Stigma and role strain – Peer leaders feared being labelled or stigmatized in the community. “Since people know that my friend tested positive with the kit I distributed, they might think I’m the HIV person now.” (Male IDI – Peer leader) |
|  | Social alignment with adolescent values – Adolescents preferred the informal, friendly approach over facility-based testing. “I feel more confident picking it from my friend than from healthcare workers who are rude.” (Female IDI, 17 years) | Distrust in sustainability and follow-up – Doubts about consistent implementation or ongoing support. “I doubt that this peer distribution programme will ever be implemented here… we don’t see any action.” (Male IDI – Peer leader) |
