# Supplementary material for: Perceived barriers and facilitators to the feasibility and acceptability of a peer-to-peer (P2P) HIV self-testing distribution model among adolescents and young people aged 15–24 years in Uganda: a qualitative study
Source: BMJ Public Health. 2026 Aug 5;4(3):e005074. doi: 10.1136/bmjph-2026-005074 (PMC13448566; doi:10.1136/bmjph-2026-005074)
Supplement: Supplementary file 4 [file bmjph-4-3-s003.doc]

**Supplemental file S3: Standards for Reporting Qualitative Research (SRQR)**

O’Brien B.C., Harris, I.B., Beckman, T.J., Reed, D.A., & Cook, D.A. (2014). Standards for reporting qualitative research: a synthesis of recommendations. *Academic Medicine, 89(9)*, 1245-1251.

| **No.** | **Topic** | **Item** | **Description** | **Notes from Manuscript** |
| --- | --- | --- | --- | --- |
| S1 | Title | Title | Concise description identifying the study as qualitative | “Feasibility and acceptability of Peer to Peer (P2P) HIV Self Testing distribution model among adolescents and young people aged 15-24 years in Uganda: a qualitative study” clearly states qualitative nature. (page 1) |
| S2 | Abstract | Abstract | Summarizes objective, methods, results, conclusions | Abstract includes introduction, objective, methods (FGDs, IDIs, thematic analysis), results (feasibility, acceptability, barriers), and conclusions. (page 2) |
| S3 | Introduction | Problem formulation | Description and significance of the problem; review of relevant theory and empirical work | Background section details HIV prevalence in Uganda, barriers to testing, importance of youth-friendly approaches, and rationale for P2P HIVST. (page 3-4) |
| S4 | Introduction | Purpose/research question | Purpose of study and specific objectives | “This study aimed at exploring the feasibility and acceptability of Peer to Peer HIV Self testing distribution model among adolescents and young people aged 15-24 years in Uganda.” (page 4) |
| S5 | Methods | Qualitative approach and research paradigm | Qualitative approach and guiding theory, research paradigm | Descriptive exploratory qualitative design; thematic analysis (Braun & Clarke, 2006); constructivist/interpretivist lens implied. (page 4) |
| S6 | Methods | Researcher characteristics and reflexivity | Researchers’ attributes, assumptions, and potential influence | Reflexivity briefly mentioned in data analysis; added notes on team discussions, reflective memos, but positionality could be expanded. (page 7) |
| S7 | Methods | Context | Setting/site, contextual factors, rationale | Conducted in three fishing communities in Wakiso District due to dynamic adolescent populations; HIVST not yet introduced to general public. (page 5) |
| S8 | Methods | Sampling strategy | Participant selection, saturation rationale | Purposive sampling through community meetings and CHEWs; sample size determined by data saturation; FGDs and IDIs conducted. (page 5) |
| S9 | Methods | Ethical issues pertaining to human subjects | Ethics approvals, consent, confidentiality | Approvals: UVRI REC, UNCST, LSHTM; written consent obtained, assent for <18; confidentiality emphasized during FGDs/IDIs. (page 6) |
| S10 | Methods | Data collection methods | Types of data, procedures, iterative process | FGDs (n=8, 68 participants) and IDIs (n=14); semi-structured guides; data collected March 2021–Feb 2022; iterative thematic exploration; triangulation of methods. (page 5-6) |
| S11 | Methods | Data collection instruments & technologies | Instruments/devices, changes | Semi-structured interview guides piloted; audio recordings; guides slightly refined iteratively; translation to English from Luganda. (page 6) |
| S12 | Methods | Units of study | Participants’ number and characteristics | 82 adolescents and young adults aged 15–24; both sexes; mix of students and out-of-school; peer leaders included; demographics reported in Table 1. (page 6) |
| S13 | Methods | Data processing | Transcription, coding, anonymization | Audio transcribed verbatim, translated, anonymized; coded manually; stored securely; team-reviewed codebook for consistency. (page7) |
| S14 | Methods | Data analysis | How themes developed, researchers involved | Hybrid thematic analysis (inductive + deductive); three researchers coded; iterative review of themes/subthemes; Braun & Clarke referenced. (page7) |
| S15 | Methods | Techniques to enhance trustworthiness | Credibility, reliability strategies | Triangulation (FGDs + IDIs); multiple coders; codebook consensus; reflective memos; representative quotes used. (page7) |
| S16 | Results | Synthesis and interpretation | Main findings, integration with prior research | Themes on feasibility (training, peer trust, logistics), acceptability (trust, autonomy, confidentiality), barriers, psychosocial burden; discussed in Results and Discussion. |
| S17 | Results | Links to empirical data | Quotes or evidence to support findings | Multiple illustrative quotes provided for each theme in Results; e.g., “Adolescents are confident among themselves… if I get it from a friend, we would even test together.” (page 8) |
| S18 | Discussion | Integration with prior work, implications | Connect findings to prior research; transferability; contribution | Discussion links to prior studies on HIVST in SSA; highlights psychosocial burden of peer distributors as novel contribution; policy/program implications suggested. (Page 17-19) |
| S19 | Discussion | Limitations | Trustworthiness and limitations | Limitations include context-specific sample, recruitment via CHEWs, potential social desirability bias, inconsistent number of FGDs, lack of health worker/caregiver perspectives. (page 17) |
| S20 | Other | Conflicts of interest | Potential influence on study | Authors declare no competing interests. (page 19) |
| S21 | Other | Funding | Funding sources and role | Funded by Wellcome Institutional Strategic Support Fund; funders had no role in design, data collection, or publication decisions. (page19) |

^a^The rationale should briefly discuss the justification for choosing that theory, approach, method, or technique rather than other options available, the assumptions and limitations implicit in those choices, and how those choices influence study conclusions and transferability. As appropriate, the rationale for several items might be discussed together.
